# New focus of Kyasanur Forest disease virus activity in a tribal area in Kerala, India, 2014

**DOI:** 10.1186/s40249-015-0044-2

**Published:** 2015-03-05

**Authors:** Babasaheb V Tandale, Anukumar Balakrishnan, Pragya D Yadav, Noona Marja, Devendra T Mourya

**Affiliations:** National Institute of Virology (NIV), 20-A, Dr Ambedkar Road, Pune, Maharashtra 411001 India; National Institute of Virology Kerala Unit, Alappuzha, Kerala India; Deputy District Medical Officer (Public Health), Malappuram, Kerala India

**Keywords:** Kyasanur Forest disease, Hemorrhagic fever, Arbovirus, Flavivirus, India, Ticks

## Abstract

**Background:**

Kyasanur Forest disease (KFD) is a febrile illness characterized by hemorrhages, and is reported endemic in the Shimoga district in Karnataka state, India. It is caused by the KFD virus (KFDV) of the family *Flaviviridae*, and is transmitted to monkeys and humans by *Haemaphysalis* ticks.

**Findings:**

We investigated a new focus of KFD among tribals in a reserve forest in Kerala state, India. A suspected case was defined as a person presenting with acute fever, headache, or myalgia. Human sera were collected and tested for KFDV RNA by real-time RT-PCR, RT-nPCR assay, and anti-KFDV IgM and IgG by ELISA. The index case was a tribal woman with febrile illness, severe myalgia, gum bleeding, and hematemesis. Anti-KFDV IgM antibody was detected in acute and convalescent sera of the index case along with IgG in the second serum. None of her family members reported fever. On verbal autopsy, two more fatal cases were identified as probable primary cases. Acute serum from a case in the second cluster was detected positive for KFDV RNA by real time RT-PCR (Ct = 32) and RT-nPCR. Sequences of E gene showed highest similarity of 98.0% with the KFDV W-377 isolate nucleotide and 100% identity with amino acid. Anti-KFDV IgM was detected in the serum of one family member of the index case, as well as in one out of 17 other tribals.

**Conclusions:**

We confirmed a new focus of KFDV activity among tribals in a reserve forest in the Malappuram district of Kerala, India.

**Electronic supplementary material:**

The online version of this article (doi:10.1186/s40249-015-0044-2) contains supplementary material, which is available to authorized users.

## Multilingual abstracts

Please see Additional file 1 for translations of the abstract into the six official working languages of the United Nations.

## Findings

Kyasanur Forest disease (KFD) is caused by the KFD virus (KFDV) of the genus *Flavivirus* and the family *Flaviviridae* [1]. It was first recognized in Karnataka state, India in 1957 [1]. The virus gets transmitted to monkeys and humans via the bite of *Haemaphysalis spinigera* ticks [2]. It causes febrile illness progressing to hemorrhages with mortality in 2–10% of the cases [2]. Kyasanur Forest disease has been endemic in the Shimoga district of Karnataka. The transmission mostly occurs during the winter and summer months [3] and for prevention, a two-dose vaccination schedule is followed in endemic areas [4]. During the last few years, the disease has spread to many new areas including Chamrajnagara (2011–12) and Tirthalli (2012–13) in Karnataka [5,6], the neighboring state Tamil Nadu, and the Wayanad district in Kerala state [7]. The first human sporadic case of KFD was reported in the Wayanad district last year [8]. We report the new focus of KFDV activity among tribals in a reserve forest in the Malappuram district of Kerala, India.

### Study area

**Figure 1:**
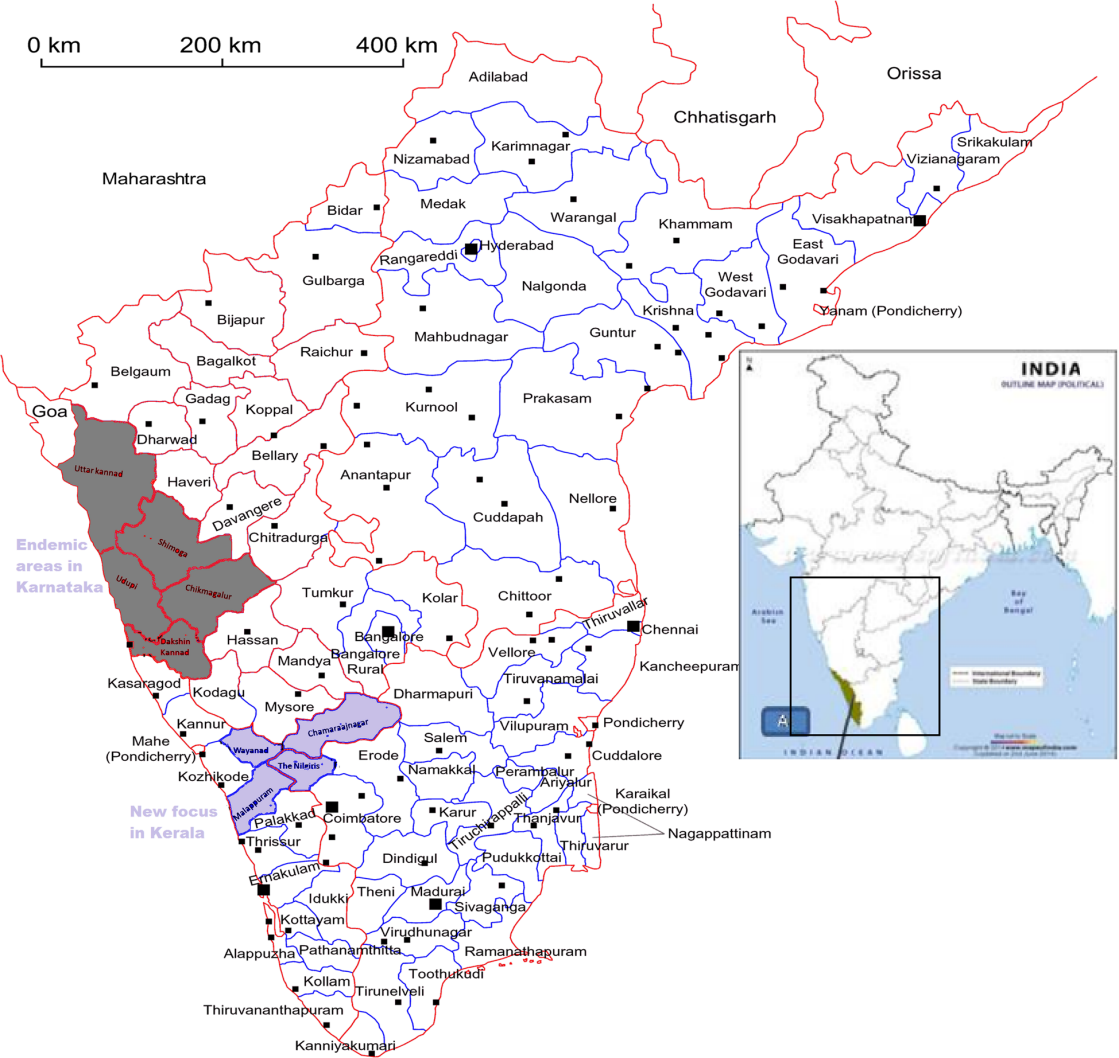
**The study area in Nedumkayam Reserve Forest in Nilambur Taluk of Malappuram district, Kerala state, India.** The study area is in Malappuram, Kerala, South India (larger panel) in India (small panel as inset). The new focus area shares borders with Karnataka in the North and Tamil Nadu in the East. The highlighted area is part of the Nedumkayam Reserve Forest in Malappuram. It forms part of the new focus along with other adjoining areas that have reported recent KFD activity as indicated in the larger panel.

The exact study area was the Nagamala hills in the Nedumkayam Reserve Forest, Malappuram, Kerala. The study area is a thick rainforest with a diverse wildlife. It is adjacent to the Nilgiri forests in Tamil Nadu and also shares borders with Karnataka (see Figure 1). The primitive nomadic tribes live inside caves or on hill slopes in the forest and do not own any domestic animals. They earn their livelihood by collecting honey and other forest items and exchanging these for required food grains and supplies. The study area is 100 plus kilometers away from the earlier endemic foci in Karnataka.

The district health authorities reported a suspected case of KFD in a woman from the Nagamala hills in the second week of May 2014. A proactive field investigation was undertaken between May 18 and 22, 2014.

### Ethics statement

The institutional human ethics committee at the National Institute of Virology (NIV), Pune approved the outbreak investigation. All adult subjects provided informed consent, and a parent or guardian of any child participant provided informed consent on the child’s behalf. Informed consent was verbal as per the national guidelines pertaining to outbreak investigations. Only those consenting for blood sampling were included in the study.

### Sample collection and testing

A suspected case was defined as a person of any age, who was a visitor or resident in the forest area, presenting with acute fever, headache, and myalgia along with exposure to ticks [3]. On verbal consent, blood samples were collected from suspected cases, family contacts, and other tribals along with the documentation of their illnesses, especially for fever and manifestations of KFD during the preceding month. Verbal autopsy of community deaths was undertaken by interviewing family members using the World Health Organization (WHO) recommended verbal autopsy questionnaire for adults [9]. We enquired about the handling of monkeys by tribal community members.

Blood samples were transported on ice to the NIV Kerala Unit, and later on dry ice for testing at the Maximum Containment Laboratory at the NIV, Pune. All human sera were tested for anti-dengue IgM and RNA detection by RT-PCR, in addition to testing for KFDV RNA using real-time RT-PCR, RT-nPCR assay, and for IgM and IgG antibody detection by anti-KFDV IgM and IgG ELISA [10]. The partial NS-5 and Pre M and E gene were also sequenced from genomic location 841–2060. Segments were amplified using single step RT-PCR as per earlier published primers on real-time RT-PCR positive serum sample. Reverse transcription was performed at 50°C for 30 minutes and PCR was performed at 94°C for two minutes, followed by 35 cycles of 94°C for 15 seconds, 50°C for 30 seconds, 68°C for two minutes, and a final extension at 68°C for 10 minutes. The PCR products were analyzed on 1.5% agarose gel electrophoresis and Ethidium bromide staining. Cyclic sequencing was carried out at PCR condition 96°C (one minute), 96°C (10 seconds), 45°C (five seconds), and 60°C (four minutes) for 25 cycles using ABI BigDye® 3.1 dye chemistry (Applied Biosystems®, Foster City, CA). These products were purified using DyeEx 2.0 kit (Qiagen) according to the manufacturer’s instructions and sequencing was performed using the ABI PRISM® 3100 automated DNA sequencer. Both DNA strands were sequenced and chromatogram data were assembled using the Sequencher 5.1 software (Accelrys Inc.) for both the reads from both the ends [11].

The details of the two separate clusters with cases and contacts are presented in Table 1. These two clusters pertain to two different families living on hills either in rock shelters or in temporary huts made of leaves. They lived almost two kilometers away from each other and were separated by two hills and valleys.

**Table 1:** **Cluster wise summary of KFD cases and contacts investigated during the study**

**Clusters**	**Case/Contact status**	**No. investigated**	**No. reported with fever**	**No. of sera**	**No. of sera positive for KFD (IgM ELISA/IgG ELISA/RNA by RT-PCR)**
*Cluster 1*					
	Index case	1	1	2	2 (IgM by ELISA) and
					1 (IgG ELISA)
	Family contacts	8	0	7	1 (IgM by ELISA)*
*Cluster 2*					
	Primary cases	2	2	0	Not applicable@
	Secondary cases	4	4	4	1 (RNA by RT-PCR)#
	Tribal contacts	17	5	17	1 (IgM by ELISA)$

*Contact did not report a history of fever.

$Contact reported a history of fever.

@ Primary cases could not be sampled as they died without medical consultation before the field investigation took place. Verbal autopsy indicated that these were suspect cases based on clinical/epidemiological history.

#The secondary case was a family member of either of the two suspected primary cases who died.

The index case in the first cluster was a tribal woman who reported a history of febrile illness for two weeks. She also reported severe myalgia, gum bleeding, and hematemesis. A blood sample was collected from her in the second week of illness. She was mildly febrile at the time of our field visit for the second blood sample collection undertaken in the late convalescent phase i.e. the fourth week of illness. However, none of her eight family members reported a history of fever.

Two more cases that succumbed to a similar illness were reported in the adjacent area, approximately one month before index case one in cluster one. They were husband and wife, aged 25 to 30 years. The husband reported febrile illness and died within a week at home without seeking medical care. The wife developed febrile illness two weeks later. She also did not seek medical care and was bedridden before death. The verbal autopsy indicated that clinical manifestations were similar to KFD. The tribals told field investigators that a monkey died one month earlier in the same area. But they did not give us the information on handling of monkeys. Among the 18 other investigated tribals, seven reported a history of fever.

All sera were negative for anti-dengue IgM ELISA and dengue RNA by RT-PCR using established in-house tests. Anti-KFDV IgM antibody was detected in high titer (P/N ratio of 6) in acute serum and low titer of IgG antibodies (P/N ratio of 2) in convalescent sera of the recovered index case, one of seven afebrile family contacts, and one more febrile tribal from the same area. Anti-KFDV IgM presence in only one asymptomatic member out of the eight family members could be due to differences in exposure or host responses. All sera were negative for anti-KFDV IgG antibody by ELISA, except the convalescent second serum of the recovered case in the first cluster. Acute serum from another suspected case was positive for KFDV RNA in real time RT-PCR with (Ct = 32) and RT-nPCR assay. The NS-5 gene partial segment showed similarity with the W-377 isolate. The partial NS-5 and Pre M and E gene (1221 bp) was also sequenced from genomic location 841–2060. Sequences of E gene showed highest identity of 98.0% with KFDV W-377 isolate. The sequence was deposited in GenBank (KP315947).

A single human case and monkey positivity was reported earlier from the adjacent Wayanad district [7,8]. We confirmed a new focus of KFDV activity with the two separate clusters of human cases (see Table 1) confirmed for the first time in a reserve forest in Kerala. The two deaths reported earlier in the same area could be considered as probable KFD cases, based on the clinical features of a verbal autopsy [9] as the clinical specimen could not be made available for testing. Also, due to the difficult terrain and tribal habitat in deep forests, follow-up of cases could not be adequately undertaken. The positivity of only one case in cluster two could be due to differences in exposure and human immune responses.

Exposure could have been by bites of nymphs of the ticks during activities in the forest. As the tribal families actually live in the forest, they are continually exposed unlike the occasional exposure encountered during visits to forests in the earlier known endemic areas in Karnataka. The forest officers, tribal health officials, and tribal leaders did not report any earlier unusual reports of monkey deaths and human illnesses, but the disease might have been established for some time in the forest without detection.

## Conclusion

The evidence of KFD circulation in three southern states of India raises serious concerns for human health. Vaccination of the tribal population was recommended and undertaken by state health authorities, along with enhanced surveillance, health education, and prevention efforts targeted at ticks or prevention of tick bites. More surveillance studies would be critical to understand the geographic extent or spread of KFD, along with its role and contribution as a disease of public health importance in south India.

## Additional file

Additional file 1:
**Multilingual abstracts in the six official working languages of the United Nations.**

## Abbreviations

KFD: Kyasanur Forest disease
KFDV: Kyasanur Forest disease virus
RNA: Ribonucleic acid
RT-PCR: Reverse transcriptase polymerase chain reaction
ELISA: Enzyme-linked immunosorbent assay
IgM: Immunoglobulin M antibody
IgG: Immunoglobulin G antibody
RT-nPCR: Reverse transcriptase nested polymerase chain reaction
