# Supplementary material for: New focus of Kyasanur Forest disease virus activity in a tribal area in Kerala, India, 2014
Source: Infect Dis Poverty. 2015 Mar 5;4:12. doi: 10.1186/s40249-015-0044-2 (PMC4351674; doi:10.1186/s40249-015-0044-2)
Supplement: Additional file 1: — Multilingual abstracts in the six official working languages of the United Nations. [file 40249_2015_44_MOESM1_ESM.pdf]

Translation of the abstract into the six official working languages of the United Nations

تركيز جديد على نشاط فيروس مرض غابة كياسانور في منطقة قبلية في كيرالا، الهند، 2014

باباصاحب ف . تندال، بلاكرشنان أنوكمر، براغيا د. يداف، د. نونا مرجا ودفندرا ت. موريا

الموجز

الخلفية: مرض غابة كياسانور(KFD) هو مرض حمّي يتصف بالنزوف، وهو متوطن في مقاطعة شيموغا في ولاية كرْنَتاكا، الهند. وهو يحدث بسبب فيروس KFD المنتمي إلى عائلة الفيروسات المصفّرة، وهو ينتقل إلى القرود والبشر عن طريق قرّاد الهرشومة *Haemaphysalis*.

الموجودات: بحثنا في التركيز الجديد لفيروس KFD بين القبائل في غابة محمية في ولاية كيرالا، الهند. وتم تحديد حالة مشتبه بها عند ظهور إصابة بحرارة مرتفعة جدا، وصداع، أو ألم عضلي. تم جمع الأمصال البشرية وفحصها بحثا عن الـ RNA الخاص بفيروس KFD بواسطة مقايسات RT-PCR، RT-nPCR في الوقت الحقيقي، والغلوبولينات المناعية M وG المضادة لفيروس KFD بواسطة مقايسة الممتز المناعي المرتبط بالإنزيم (ELISA). كانت الحالة المؤشرة لامرأة من القبيلة مصابة بمرض حمّي، ألم عضلي شديد، ونزف من اللثة، ونفث الدم. تم تحري الغلوبولين المناعي M المضاد لفيروس KFD في أمصال الإصابات الحادة والناقهة للحالة المؤشرة إلى جانب الغلوبولين المناعي G في المصل الثاني. لم يبلغ أي من أفراد أسرتها عن حدوث حمّى. عند وضع تقرير الوفاة اللفظي، أمكن تحديد حالتين قاتلتين أخريين كحالات أولية محتملة. ثبتت إيجابية الـ RNA الخاص بفيروس KFD في مصل حالة حادة من المجموعة الثانية بواسطة مقايسات RT-PCR (إجمالي عدد الحالات= 32) وRT-nPCR في الوقت الحقيقي. أظهرت تسلسلات الجين E التشابه الأكبر بنسبة 98.0% مع نيوكليوتيد عزلات فيروس KFD W-377 وبنسبة 100% مع الحمض الأميني. وتم تحري الغلوبولينات المناعية M المضادة لفيروس KFD في مصل أحد أفراد أسرة الحالة المؤشرة، إضافة إلى واحد من 17 قبيلة أخرى.

الاستنتاجات: تأكدنا من تركيز جديد لنشاط فيروس KFD بين القبائل في غابة محمية في مقاطعة مالابورام كيرالا،الهند.

Translated from English version into Arabic by Lina SM, through

TRANSLATORS WITHOUT BORDERS

卡萨诺尔森林病病毒在印度的喀拉拉邦部落地区出现新热点

Babasaheb V Tandale, Balakrishnan Anukumar, Pragya D Yadav, Dr Noona Marja and Devendra T Mourya

摘要

引言：卡萨诺尔森林病（KFD）是一种发热性疾病，其特点是伴有出血。KFD 在印度卡纳塔克邦希莫加区流行，由黄病毒科的 KFD 病毒引起，经蜱传播至猴子和人类。

结果：本研究发现，卡萨诺尔森林病在印度喀拉拉邦自然保护区森林的部落出现新热点，患者出现急性发热、头痛或肌痛即诊断为疑似病例。收集患者血清，使用 RT-PCR 和 RT-nPCR 检测 KFD 病毒，ELISA 检测抗 KFD 病毒的 IgM 和 IgG 抗体。第 1 组病例中的 1 例索引病例是一名部落女性有发热症状伴随严重肌痛、牙龈出血和咯血。该病例在急性期和恢复期均检出抗 KFD 病毒的 IgM 抗体，IgG 抗体只在恢复期被检出。其家庭成员未报告发烧。尸检确定了两例已死亡病例可能为原发病例。第 2 组病例中有 1 例急性期血清进行 RT-PCR（Ct=32）和 RT-nPCR 检测，其 KFD 病毒 RNA 均为阳性。基因测序结果显示，其与 KFD 病毒 W-377 株的核酸相似性高达 98%，氨基酸相似性为 100%。在索引病例的 1 个家庭成员的血清中发现抗 KFD 病毒 IgM 抗体，并且该家庭成员未曾居住在自然保护区森林所属的 17 个部落中。

结论：本研究证实在印度喀拉拉邦的默勒布勒姆区的自然保护区森林里的部落之间出现一个新的卡萨诺尔森林病病毒活动热点。

Translated from English version into Chinese by Zheng Qi, through

CHINESE CENTER FOR DISEASE CONTROL AND PREVENTION
NATIONAL INSTITUTE OF PARASITIC DISEASES

Nouvel éclairage sur l'activité du virus de la maladie de la Forêt de Kyasanur dans la région tribale de Kerala, Inde, en 2014.

Babasaheb V Tandale, Balakrishnan Anukumar, Pragya D Yadav, Dr Noona Marja and Devendra T Mourya

Résumé

Contexte: la Maladie de la Forêt de Kyasanur (MFK) est une maladie fébrile caractérisée par des hémorragies, et déclarée endémique dans le district de Shimoga, dans l'Etat de Karnataka en Inde. Elle est causée par le virus MFK (VMFK) de la famille des Flaviviridae, et elle est transmise aux singes et aux humains par les tiques *Haemaphysalis*.

Résultats: Nous avons orienté une nouvelle recherche de la MFK parmi les membres tribaux dans une réserve forestière de l'Etat de Kerala, en Inde. Un cas a été défini comme suspect lorsqu'une personne présente une forte fièvre, des maux de tête, ou des douleurs musculaires. Du sérum humain a été recueilli et examiné en recherche du VMFK RNA par une analyse en temps réel RT-PCR, RT-nPCR, et de l'anti-VMFK IgM et IgG par ELISA. Le cas de référence était une femme de la tribu présentant une maladie fébrile, de fortes douleurs musculaires, un saignement des gencives, et une hématémèse. Un Anticorps Anti-VMFK IgM a été détecté dans le sérum en phase aigüe et de convalescence du cas de référence, avec IgC dans le second sérum. Aucun des membres de sa famille n'a rapporté de fièvre. En autopsie verbale, deux autres cas mortels ont été identifiés comme probables cas primaires. Dans le second groupe, du sérum en phase aigüe a été détecté positif au VMFK RNA par analyse en temps réel RT-PCR (Ct=32) et RT-nPCR. Des séquences du gène E ont montré la plus grande similitude de 98.0% avec le VMFK W-377 isolat nucleotide et une identité de 100% avec les acides aminés. Un Anti-VMFK IgM a été détecté dans le sérum d'un membre de la famille du cas de référence, ainsi que dans 1 sur 17 (un sur dix-sept) autres membres tribaux.

Conclusions: Nous confirmons un nouvel éclairage de l'activité du VMFK parmi les membres tribaux d'une réserve forestière dans le District Malappuram de Kerala, en Inde.

Translated from English version into French by Suzanne Assenat, through

TRANSLATORS
WITHOUT BORDERS

Новый очаг вспышки вируса Кьясанурской лесной болезни обнаружена на территории проживания племени в Керала, Индии, 2014 г.

Бабасахеб В Тандале, Балакришнан Анукумар, Прагья Д Йадав, Доктор Нуна Маржа и Девендра Т. Мурья

Аннотация

Справочная информация: кьсанурская лесная болезнь (КЛБ) острая инфекционная природно-очаговая болезнь из группы геморрагических лихорадок, распространенная в районе Шимога, штата Карнатака, Индия. Возбудитель болезни - вирус КЛБ рода Flaviviridae, передается обезьянам и человеку через укус клещей *Haemaphysalis*.

Анализы: нами был исследован новый очаг болезни КЛБ среди жителей племен, населяющих лесной заповедник в штате Керала, Индия. Подозрительный случай был выявлен у больного с острой лихорадкой, головной, или мышечной болью. Была взята сыворотка и проверена на РНК КЛБ при помощи ПЦР в реальном времени с реакцией обратной транскрипции, и n-гнездовой ПЦР с применением обратной транскрипции, а антивирус иммуноглобулинов класса G и M КЛБ иммуно-ферментным анализом. Носителем инфекции оказалась женщина, член одного из племен, у нее было лихорадочное заболевание, сильная мышечная боль, кровоточивость десен и рвота кровью. Антивирусы иммуноглобулина класса M к КЛБ были обнаружены в пробах, взятых в острый период заболевания и период выздоровления у больной женщины, наряду с иммуноглобулинами класса G во время повторного забора проб. У других членов ее семьи не было лихорадки. Опрос членов ее семьи и близких показал два смертельных случая, которые и определили как возможные первые случаи заболевания. Результаты анализов проб, взятых в период острого заболевания во время второго группового анализа, оказались положительными на наличие РНК КЛБ при проведении ПЦР в реальном времени с реакцией обратной транскрипции(Ct=32) и n-гнездовой ПЦР с применением обратной транскрипции. Было обнаружено очень близкое сходство в 98, 0% последовательности расположения генов E показала с несвязанным нуклеотидом W-377 КЛБ, и 100%-ное совпадение с аминокислотой. Иммуноглобулин класса G анти-вируса КЛБ был обнаружен в сыворотке члена семьи носителя инфекции, а также у одного из других 17 племен.

Заключение: подтвержден новый очаг вспышки вируса КЛБ среди жителей племен, населяющих заповедные леса Малаппурам, штат Керала, Индия.

Translated from English version into Russian by Gulnara Krokhaleva, through

TRANSLATORS WITHOUT BORDERS

Nuevo foco de la actividad del virus de la Enfermedad de la selva de Kyasanur en el área tribal en Kerala, India, 2014

Babasaheb V Tandale, Balakrishnan Anukumar, Pragya D Yadav, Dr Noona Marja y Devendra T Mourya

Resumen

Antecedentes: La Enfermedad de la selva de Kyasanur (KFD) es una enfermedad febril caracterizada por hemorragias, y se reporta como endémica en el distrito Shimoga en el estado de Karnataka, India. Es causada por el virus KFD (KFDV) de la familia Flaviviridae, y es transmitida a monos y humanos por garrapatas *Haemaphysalis*.

Hallazgos: Hemos investigado un nuevo foco de KFD entre miembros de las tribus en una reserva forestal en el estado de Kerala, India. Un caso sospechoso se definió como la presentación en una persona de fiebre aguda, dolor de cabeza o mialgia. Se recolectaron sueros humanos y se los analizó buscando el ARN de KFDV usando RT-PCR en tiempo real, RT-nPCR y anti-KFDV IgM y IgG mediante ELISA. El caso índice fue el de una mujer de la tribu con enfermedad febril, mialgia grave, sangrado en las encías y vómitos de sangre (hematemesis). Fue detectado el anticuerpo anti-KFDV IgM en los sueros agudo y convaleciente del caso índice en conjunto con IgG en el segundo suero. Ningún miembro de la familia de la mujer presentó fiebre. En la autopsia verbal, fueron identificados otros dos casos fatales como casos primarios probables. El suero agudo de un caso en el segundo grupo fue detectado como positivo en el ARN de KFDV por RT-PCR (Ct=32) en tiempo real y RT-nPCR. La secuencias del gen E mostraron la más alta similitud de un 98,0% con el nucleótido de KFDV W-377 aislado y un 100% de identidad con aminoácidos. Fue detectado el anti-KFDV IgM en el suero de un miembro de la familia del caso índice, como así también en uno de 17 otros miembros de la tribu.

Conclusiones: Hemos confirmado un nuevo foco de la actividad del KFDV entre los miembros de la tribu en una reserva forestal en el distrito de Malappuram de Kerala, India.

Translated from English version into Spanish by two2tango, through

TRANSLATORS
WITHOUT BORDERS
